# Fiber Bragg Grating Wavelength Drift in Long-Term High Temperature Annealing

**DOI:** 10.3390/s21041454

**Published:** 2021-02-19

**Authors:** Dan Grobnic, Cyril Hnatovsky, Sergey Dedyulin, Robert B. Walker, Huimin Ding, Stephen J. Mihailov

**Affiliations:** National Research Council Canada, 100 Sussex Drive, Ottawa, ON K1A 0R6, Canada; Kyrylo.Hnatovsky@nrc-cnrc.gc.ca (C.H.); Sergey.Dedyulin@nrc-cnrc.gc.ca (S.D.); Robert.Walker2@nrc-cnrc.gc.ca (R.B.W.); Huimin.Ding@nrc-cnrc.gc.ca (H.D.); Stephen.Mihailov@nrc-cnrc.gc.ca (S.J.M.)

**Keywords:** high temperature, FBG, sensing, fiber Bragg gratings, wavelength drift

## Abstract

High-temperature-resistant fiber Bragg gratings (FBGs) are the main competitors to thermocouples as sensors in applications for high temperature environments defined as being in the 600–1200 °C temperature range. Due to their small size, capacity to be multiplexed into high density distributed sensor arrays and survivability in extreme ambient temperatures, they could provide the essential sensing support that is needed in high temperature processes. While capable of providing reliable sensing information in the short term, their long-term functionality is affected by the drift of the characteristic Bragg wavelength or resonance that is used to derive the temperature. A number of physical processes have been proposed as the cause of the high temperature wavelength drift but there is yet no credible description of this process. In this paper we review the literature related to the long-term wavelength drift of FBGs at high temperature and provide our recent results of more than 4000 h of high temperature testing in the 900–1000 °C range. We identify the major components of the high temperature wavelength drift and we propose mechanisms that could be causing them.

## 1. Introduction

The majority of present-day industrial processes require complex monitoring and control. Over the last decade, the demand for sensors having high accuracy, repeatability, reliability and the capability to be easily distributed has increased significantly. While there is a great variety of sensors which exploit different principles and generally fulfill this demand, many sensors will simply fail or will not perform according to the requirements when used in high temperature processes and extreme environments, such as in the presence of intensive ionizing radiation, strong electromagnetic field, corrosive ambient etc.

Traditionally, the majority of temperature measurements for applications in the 600–1200 °C temperature range were done with various thermocouples [1,2]. Thermocouples use a different physical principle to measure temperature, namely, they measure changes in the electromotive force between two dissimilar metals as a function of temperature. The main disadvantages of thermocouples that currently limit their use are their large size (the electromotive force is essentially generated by thermal gradients along the entire length of the thermocouple), redistribution of impurities/dopants along the thermocouple length at high temperatures (the electromotive force is altered during the measurements) and sensitivity to electromagnetic interference and ionizing radiation [1]. These drawbacks can potentially be addressed by fiber optic sensors (FOSs). Being small, insensitive to electromagnetic radiation, resistant to corrosive environments, capable of multiplexing and relatively cheap to manufacture, FOSs are, at a first glance, ideally suited to perform sensing in harsh environments as those found in fossil fuel extraction and processing, nuclear power plants, combustion and gas turbine engines. Indeed, there are experiments described in the literature that involve simultaneous measurements by FOSs and thermocouples [3,4,5] and show similar temperature measurement results.

A relatively large assortment of FOSs, ranging from simple fiber devices that exploit Rayleigh [6], Brillouin and Raman [7] scattering effects to fiber Bragg gratings (FBGs), sophisticated Fabry-Pérot sapphire interferometers [8] and contact pyrometers [9], has been developed to measure high temperature. For very high temperature range (900–1900 °C) sapphire FBGs are being developed [10]. However, based on the published results related to the deployment of FOSs beyond the lab environment, the main candidates for this purpose rely on the FBG technology. Specifically they are:

(1) Regenerated fiber Bragg gratings (RFBG) made by using ultraviolet (UV) lasers and the phase mask technique [11],
(2) FBGs made by using femtosecond lasers and the phase mask technique (Type II FBGs) [12,13].
(3) FBGs made by using femtosecond lasers and the point-by-point (PbP) technique (PbP FBGs) [14].

All of the above types of FBGs (i.e., RFBGs, Type II FBGs and PbP FBGs) have good thermal stability up to 1000 °C, whereas the classical FBGs (i.e., Type I UV laser induced FBGs) are almost completely erased at 700 °C. RFBGs were discovered in 1997 [15] as a result of high temperature annealing of hydrogen-loaded fibers containing Bragg gratings that were inscribed with UV light using the phase mask technique. It was observed that after the erasure of the seed FBG, further heating at higher temperatures generated a new grating structure at a longer Bragg resonance wavelength. The index modulation of this new grating was significantly smaller than that of the original seed grating by about an order of magnitude. It was initially proposed that fluorine dopant, which was present alongside germanium dopant in the fiber core, was responsible for the interesting effect of regeneration. However, it was later shown that regeneration could take place without the presence of fluorine [11]. 

The inscription in silica fibers of high temperature resistant FBGs using infrared (IR) ultrafast radiation and the phase mask technique was demonstrated for the first time in 2003 [16]. The application of the phase mask technique was similar to that used to fabricate RFBGs with UV radiation, i.e., placing a phase mask into the laser beam to create an interference pattern that photo-induces periodic structural changes in the fiber core. Soon afterwards, in 2004, a periodic Bragg grating pattern was successfully inscribed into the fiber core using ultrafast IR radiation and the PbP technique, in which case the IR pulses were tightly focused inside the fiber using a microscope objective and an FBG was produced by precisely translating the fiber with respect to the fixed laser focus [17].

The idea underlying the use of FBGs as temperature sensors is straightforward. A FBG temperature sensor is a narrow-band interference filter whose reflection spectrum is centered at a characteristic wavelength which is called the Bragg wavelength *λ_B_*. The fulfillment of the Bragg condition leads to a simple relation between *λ_B_* and the parameters defining the FBG structure, namely the effective refractive index of the core *n_eff_* and the period of the refractive index modulation Λ*_G_*:

λ*_B_* = 2·*n_eff_* ·Λ*_G_* (1)

As both *n_eff_* and Λ*_G_* are functions of temperature, *λ_B_* is also correlated with the ambient temperature. Type II FBGs, for example, demonstrated near-linear wavelength drifts with temperature of 16.8 pm/ °C in the 800–1000 °C temperature range [18].

Using this basic principle, high temperature resistant FBGs were deployed in a considerable number of applications. RFBGs were used to monitor the temperature of a melt during metal processing [19], the temperature of a turbo engine exhaust [20], during structural fire testing [21] and for measurement of the temperature distribution inside the substrate during a MCVD process [22]. An array of six RBFGs was used to thermally map the exhaust of a gas turbine [23] and 24 RFBGs incorporated into six fibers were used to monitor the temperature profile of a catalytic fixed-bed tubular reactor for more than two years [24]. 

Type II FBG arrays have been used in several applications: for monitoring temperature gradients of the flame tube of a low emission burner [25], to monitor an entrained flow gasifier [18] and an oxy-fuel fluidized bed combustor [26]. Type II FBGs fabricated inside an air-hole fiber were used to measure pressure in a high temperature ambient environment (800 °C) [27]. 

A number of tests were also performed in nuclear installations. As an example, RFBGs were placed in liquid sodium at 500 °C for high temperature monitoring of a sodium-cooled fast nuclear reactor [28]. Four fibers with 11 RFBGs inside each were used as temperature sensors in the plasma-facing components in a Tokamak installation [29]. Two Type II FBGs were used to measure temperature during physical simulations of an accident in a transient reactor test facility [30].

The above published results confirm that FBG sensors can potentially replace thermocouples in some applications and, moreover, improve the quality of temperature measurements by leveraging the unique advantages of FOSs. However, at temperatures approaching 1000 °C, the silica glass of which the fiber is made of, starts changing its properties, which could potentially lead to degradation in the sensor’s performance. The first experiments to establish the survivability of FBG structures at high temperature mainly concentrated on monitoring the reflectivity of the FBGs under test [31]. The next step was to test FBGs as integral parts of temperature sensing instruments that would be used, stored and re-used. For this purpose the ambient temperature was cycled between room and the target temperature and, in general, the results looked promising as little or no hysteresis was detected [32], at least over the relatively short dwell time of a few hours at high temperature. Many high temperature applications, however, require long-term monitoring, sometimes at the level of years. In this scenario, in addition to monitoring changes in the magnitude of the FBG reflectivity, the stability of the Bragg wavelength also needs to be evaluated over extended periods of time.

Studying the Bragg wavelength drift of high temperature resistant FBGs (i.e., RFBGs, Type II FBGs and PbP FBGs) at a constant temperature (i.e., around 1000 °C) is the main subject of this paper. It summarizes previously reported experiments and presents our recent tests performed with Type II FBGs. If no stress is applied to the fiber, the Bragg wavelength drift at a constant temperature (see Equation (1)) will be caused by changes in *n_eff_* and Λ*_G_* due to several effects such as dopant diffusion from the core, devitrification (crystallization) of the glass, and release of stresses that are built into the fiber during the fiber manufacturing process. Hence, a more specific aim of this study is to look at the most probable mechanisms of the Bragg wavelength drift in silica fibers and suggest ways to mitigate their effect. This becomes especially important because the initial proof-of-concept successes with 1000 °C-resistant sapphire FBGs [33,34,35] and sapphire-derived fibers [36] were not followed by real-life applications. The use of silica-based FOSs comprising high temperature resistant FBGs thus remains the only practical option for distributed high temperature sensing.

## 2. High Temperature FBG Structures

According to Equation (1), the Bragg wavelength of an FBG (*λ_B_*) is a function of the FBG’s period (Λ*_G_*) and the modal effective refractive index (*n_eff_*). The latter is determined both by the fiber and the refractive index modulation inside the FBG, which in turn depends on the internal structure of the FBG. Therefore, one of the first questions to ask is whether the Bragg wavelength drift at high temperature is caused by transformations occurring in the optical fiber itself or in the FBG internal structure, or both. In order to answer this question the FBG internal structure should be known. Below we list the available information on the FBG internal structure.

### 2.1. RFBG Structure

The RFBG fabrication process is relatively straightforward and extensively discussed in the literature [37]. Typically, a FBG is inscribed in a hydrogen-loaded fiber with UV lasers and a phase mask that is afterward annealed at a certain temperature (500 °C–1000 °C). This annealing results in the complete erasure of the original FBG (i.e., seed FBG) and the subsequent growth of a new FBG (i.e., regenerated FBG). While the fabrication process is fairly simple, an explanation for the regeneration effect has proven to be much more difficult to find. Over the years, the mysterious origin of RFBGs has attracted considerable interest and a number of models have been created to explain it only to find a contradiction in the explanation as new experimental results were produced. According to an excellent review on the history of RFBGs [38], they were initially referred to as Chemical Composition Gratings [15] because it was thought that the grating structure was created by the elimination of fluorine at high temperature from the sites in the fibers core where OH was produced under the UV radiation. Later, this explanation evolved into a stress model independent of the presence of fluorine [39] where UV radiation-induced OH groups enhanced tensile stress at the core-cladding interface that would eventually turn into compressive stress at high temperature, with crystallization also playing a role. A later model [40] suggests that the compressive crystallization at high temperature is triggered by the release of stress due to the penetration of molecular hydrogen. While not based on a well-defined theory, the model presented above suggested that stress/densification strongly affects the behavior of RFBGs.

### 2.2. Type II FBG Structure Made with an IR Femtosecond Laser and a Phase Mask

Type II FBGs are fabricated with an ultra-high-intensity interference pattern at the core of the fiber. The exposure results in Type II modifications which are structural changes in the fiber glass resulting from the glass interaction with intense ultrafast radiation having peak intensities on the order of 10^13^ W/cm^2^. Under specific irradiation conditions, dielectric breakdown occurs in the form of self-organized nanogratings [41], which are planar form-birefringent structures that survive temperatures up to 1000 °C. In 2017 [42], using scanning electron microscopy (SEM), nanogratings were found inside Type II FBGs inscribed with multiples femtosecond pulses. While light-induced nanogratings are highly regular structures, Type II modification in bulk dielectrics is generally represented by irregular nanoporous structures having a significantly different refractive index compared with the surrounding unmodified material [43]. It is also expected that these nanopores and nanopore-containing regions induce high stress in the material. Whether Type II FBGs can be built of planes containing nanoporous material, or single micropores (microvoids), or a combination thereof, remains an open question and is a subject of our current studies.

### 2.3. PbP FBG Structure

The PbP inscription technique consists of tight focusing of femtosecond pulses in the core of the fiber while maintaining a very good control of the relative position between the core of the fiber and the focus using precision air bearing stages. Each exposure creates a single period of the grating and the grating is created in a “step and repeat” approach [44]. The internal structure of PbP FBGs seems to be the simplest of all the FBGs presented. The high peak intensity pulses create microvoids surrounded by a high density shell in the irradiated fiber material [45]. The refractive index of the microvoid is unity, while the refractive index of the densified material surrounding it must be higher than that of the pristine fiber material. As a consequence, the local effective index of the fiber containing PbP FBGs can become lower than that of the pristine fiber material, which may affect the wavelength drift mechanism.

Although the fabrication techniques of high temperature resistant FBGs are very different, it appears that their general structure can be modelled in terms of successive areas of high and low density with built-in stresses, so some similarity in the behavior should be expected A high temperature ambient environment can cause changes in these structures due to stress relaxation and mass transfer, which in turn will contribute to the wavelength drift experienced by the FBGs.

### 2.4. Optical Fiber Structure

An optical fiber is a glass filament (typically ~125 µm in diameter in the experiments presented in this paper) having a core of a much smaller diameter that confines the light, preventing it to leak to the external region called cladding. The manufacturing process of optical fiber consist of drawing the fiber from a preform heated in a furnace to the glass softening temperature. When the fiber exits the furnace it starts cooling from high (above 1700 °C) temperature to room temperature after which it is coated and wound on a drum. Besides the intended effect of creating the core/cladding refractive index structure, the drawing and cooling processes result in a pattern of stresses (tensile and compressive, both radial and longitudinal) frozen into the fiber after the drawing process is finished [46]. The frozen-in stresses, also known as residual stresses, are classified as (1) thermal stresses if the core and cladding have different thermal expansion coefficients and (2) mechanical stresses if different parts of the fiber have different viscosities during the drawing process. In SMF-28 fiber the pure silica cladding will always be more viscous and solidify first, resulting in a strong compression of the less viscous core–the compression that will stay in place at room temperature. Annealing of the fiber at temperatures below the transition temperature [46] will gradually reduce frozen-in stress and, as a result, slowly change the effective refractive index of the core through the photo-elastic effect.

## 3. Literature Review

In this section, we present a summary of experiments performed to study high temperature wavelength drift of: (1) RFBGs, (2) Type II FBGs and (3) PbP FBGs. Experiments on high temperature annealing of other structures such as (i) ablation FBGs in photonic crystal fibers (PCF), (ii) FBGs in chiral fibers, (iii) Fabry-Pérot fiber cavities and (iv) nanogratings in bulk glasses are also included in the discussion as they provide information about possible mechanisms, such as dopant diffusion or stress relaxation, that may cause a wavelength drift of FBGs. 

### 3.1. Regenerated Gratings

**Figure 1:**
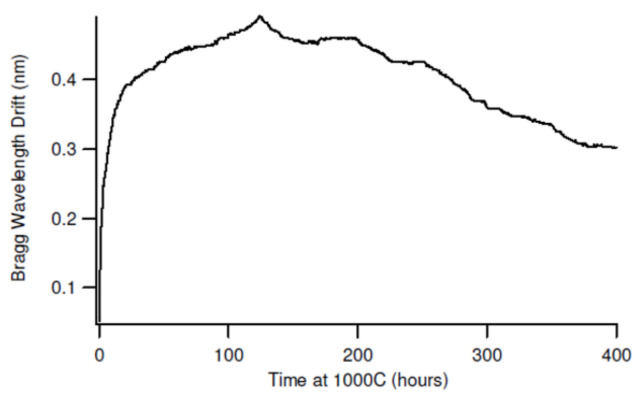
Bragg wavelength drift of a RFBG (reproduced from [47]).

The long-term wavelength stability of RFBGs at high temperature was presented for the first time in 2005 [47]. During the heating-up period the original FBGs dissipated at ~880 °C and the respective RFBGs started to emerge at ~913 °C. After the start of the regeneration process, the Bragg wavelength of the gratings experienced a drift toward longer wavelength (red drift) while the reflectivity of the regenerated gratings increased. By the time the temperature reached 1000 °C, the RFBG reflectivity increased to a maximum value corresponding to ~20 % of the initial reflectivity of the seed grating and then decreased asymptotically to reach a stable value corresponding to ~7% of the initial reflectivity after ~5 h. The gratings were further tested at 1000 °C for ~400 h and the Bragg wavelength was monitored (see Figure 1) [47]. 

During the test, the Bragg wavelength experienced a red drift of ~480 pm during the first 125 h of annealing and then a 190 pm blue drift (i.e., drift toward shorter wavelengths) during the remaining 270 h. The initial red drift of 480 pm was likely the result of the regeneration process. The majority of the red drift took place in the first 25 h at a rate of ~16 pm/h. Looking for an explanation for the post-regeneration drift at 1000 °C, the authors examined samples of the annealed fiber for signs of devitrification using an optical microscope but did not find any crystalline regions in the fiber.

**Figure 2:**
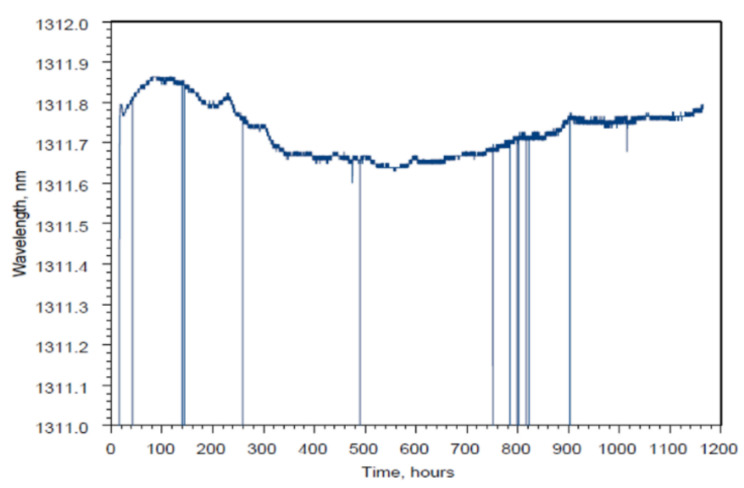
Bragg wavelength drift of a RFBG at 1000 °C (reproduced from [48]).

A much longer annealing experiment at 1000 °C was performed on RFBGs in 2011 [48]. The authors do not mention how many gratings were used but two gratings survived to 1000 °C and were then monitored for 1200 h. As presented in Figure 2, the Bragg wavelength underwent a ~100 pm red drift in the first 50 h of annealing at 1000 °C, then a ~200 pm blue drift in the next 500 h, almost no drift during the subsequent 400 h, a ~100 pm red drift again over the following 300 h and almost no drift during the final stage of the experiment. It is one of the most complex wavelength drift patterns reported so far, which seems to suggest that multiple processes were at play. The authors offered no explanation for the Bragg wavelength evolution but it is worth noting that the gratings were packaged inside ceramic tubes which could have applied stresses to the fiber.

**Figure 3:**
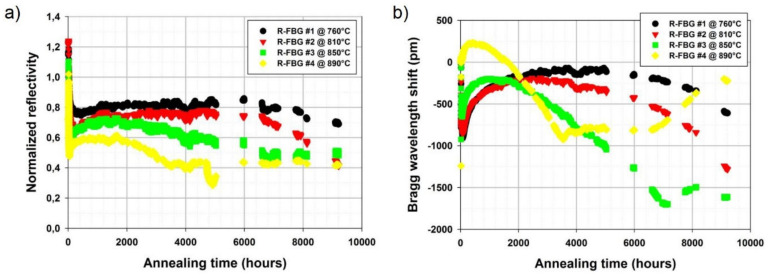
Long-term annealing data of RFBGs. (**a**) normalized reflectivity and (**b**) Bragg wavelength drift (reproduced from [49]).

The longest high temperature test performed with RFBGs was reported in [49] when four gratings created in H_2_-loaded SMF-28-type fiber (i.e., SMT-A1310H germano-silicate fiber from OFS Company, Norcross, GA, USA) were annealed for 9000 h at different temperatures between 700 °C and 900 °C. The annealing temperature of each RFBG was continuously monitored by an individual thermocouple giving 760 °C, 810 °C, 850 °C and 890 °C. As can be seen in Figure 3a, a rapid decrease followed by stabilization of reflectivity was observed for all the RFBGs during the first 50-200 h of annealing at the respective temperatures. After the initial phase of annealing, the RFBG reflectivities decreased very slowly until the end of the experiment, with the RFBGs maintained at 850 °C and 890 °C being the most stable. Figure 3b shows the evolution of the Bragg wavelengths of the four RFBGs during the annealing test. Even though the data pertaining to Figure 3b are self-explanatory, some trends are still worth mentioning. The Bragg wavelengths of the RFBGs at 760 °C and 810 °C experienced a ~700 pm red drift in the first 2000 h, remained relatively constant for another ~2000 h and then blue-drifted by respectively ~500 pm and ~1100 pm in the remaining hours. 

Some correlation between the evolutions of the Bragg wavelengths and the reflectivities of the RFBGs at 760 °C and 810 °C is also observable. The behavior of the RFBGs at higher temperatures was more erratic. For example, the Bragg wavelength of the RFBG at 890 °C experienced a red drift of ~500 pm in the first 500 h, then a blue drift of ~1200 pm in the next 3000 h, remained stable for further ~3000 h and finally experienced a red drift of ~700 pm. It is also to be noted that variations in the behavior the RFBGs became stronger and faster as the annealing temperature increased.

**Figure 4:**
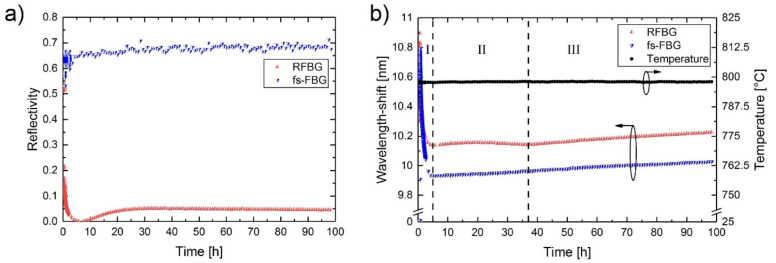
Simultaneous annealing study of a PbP FBG and a RFBG at 800 °C. (**a**) reflectivity and (**b**) Bragg wavelength drift (reproduced from [50]).

In 2018, a simultaneous annealing in a tube furnace at 800 °C of a PbP FBG made in SMF-28 and a RFBG also made in SMF-28 was performed [50] (see Figure 4). The 800 °C isothermal annealing process is split in three phases. Phase I begins when the furnace temperature reaches 800 °C and ends when the Type I seed FBG is erased and a new regenerated grating starts to emerge. During Phase I (Figure 4b) the Bragg wavelength of the Type I FBG drifted ~700 nm toward shorter wavelength during grating erasure. Phase II was characterised by almost no change in the Bragg wavelength and lasted from the emergence of the regenerated grating until the beginning of a Bragg wavelength red drift. Phase II is also coincident with the growth of the regenerated grating up to a 5% reflectivity and the stabilisation of its structure. In Phase III the Bragg wavelength red-drifted in a linear fashion at a rate of about 1.2 pm/h. Interestingly, the PbP FBG also showed a uniform red wavelength drift during Phase II with a similar rate, i.e., ~1 pm/h. The very similar behavior at 800 °C of two gratings with very different structure implies that, at least for the first 100 h of annealing at this temperature, the fiber rather than the grating structure determines the wavelength drift.

### 3.2. FBGs Witten Point by Point using a Femtosecond Laser(PbP FBGs)

**Figure 5:**
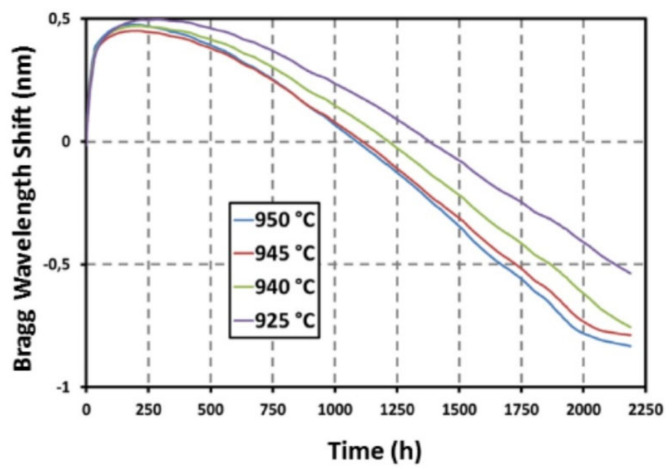
Bragg wavelength drift of 4 PbP FBGs annealed at high temperature (reproduced from [51]).

Results of a 2200-h annealing test performed at 920–950 °C with four PbP FBGs were published in 2018 [51]. Four PbP FBGs were positioned at different location in a furnace so that the local temperatures were 925 °C, 940 °C, 945 °C and 950 °C. The evolutions of the Bragg wavelengths, as shown in Figure 5, show a similar pattern for all the FBGs under test and the magnitude of the effect is well correlated with the local temperature of each FBG. All the FBGs drifted toward longer wavelengths in the first 200 h by ~500 pm, with the largest drift (400 pm) taking place in the first 25 h at a rate of 16 pm/h. After a period of 400 h of slow change (less than 100 pm), all the FBGs experienced a blue drift at a rate that was well correlated with the temperature. The blue drift rates of the FBGs at 945 °C and 950 °C were the highest, at ~0.75 pm/h and ~0.8 pm/h, respectively. The slowest blue drift rate of 0.2 pm/h was experienced by the grating placed at the lowest temperature (i.e., 925 °C) while the grating placed at the highest temperature (i.e., 940 °C) drifted at an intermediate rate of ~0.55 pm/h. The authors speculated that the changes in the micro-void pattern and/or the densified shell around the micro-voids may have been responsible for the wavelength drift. The authors also drew attention to the fact that the wavelength drift pattern presented in Figure 5 is rather similar to that of the RFBGs presented in Figure 3 above [49]. Indeed, the comparison between the RFBG annealed at 890 °C [49] and the PbP FBG that was annealed at 925 °C shows very similar blue drift rates of 0.3 pm/h and 0.2 pm/h, respectively.

### 3.3. FBGs Made with a Phase Mask and a Femtosecond Laser (Type II FBGs)

**Figure 6:**
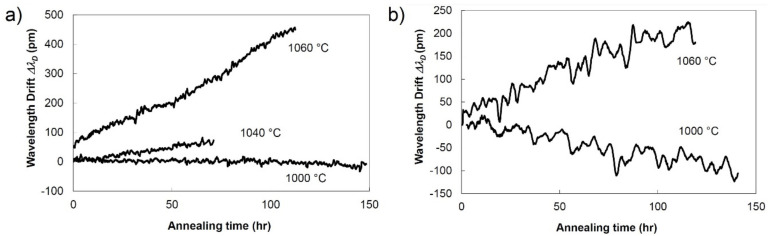
Bragg wavelength drift of a Type II FBG made using 1.6-ps pulses (**a**) in SMF-28 (**b**) in pure core silica fiber (reproduced from [52]).

The earliest report of the wavelength drift at 1000 °C of Type II FBGs made with infrared ultrafast radiation and a phase mask was presented in [52]. SMF-28 and pure-silica core single mode fiber were exposed with 800 nm, 1.6 ps pulses through a 4th-order phase mask and then heated in a tube furnace in steps of 100 °C every hour until they reached 1000 °C. They were kept at that temperature for 150 h and the Bragg wavelength was recorded every 8 min. The evolution of the Bragg wavelength of the grating in SMF-28 is presented in Figure 6a. At 1000 °C there is practically no drift, but at 1040 °C there is already a red drift rate of ~1 pm/h. At a higher temperature (i.e., 1060 °C) the wavelength drift rate becomes very high (4 pm/h). The drift of the pure silica core fiber with a fluorine-doped cladding is quite different (Figure 6b). At 1000 °C its Bragg resonance was blue-drifting at ~1pm/h rate, while at 1060 °C there was a red drift rate of 2 pm/h. Interestingly, no red drift was recorded at the beginning of the annealing although the single difference with the other test presented in this work is the 1.6 ps pulse duration used for inscription.

**Figure 7:**
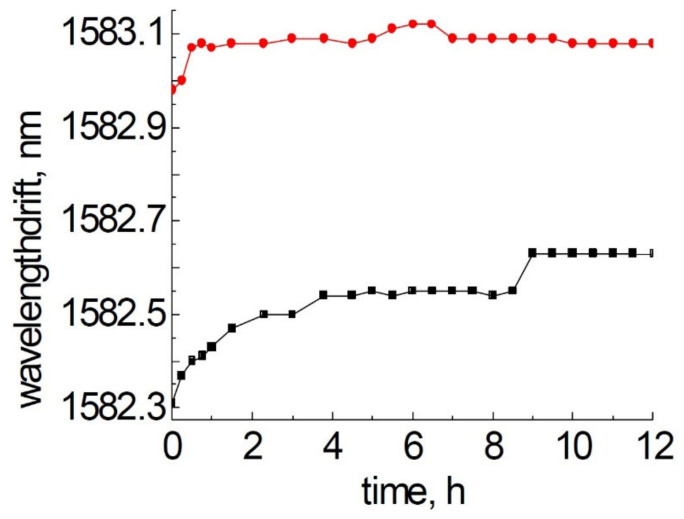
Bragg wavelength drift of Type II FBGs in SMF-28 (black squares) and H_2_-loaded SMF-28 (red circles) at 1000 °C, data reproduced from [53].

A relatively short (i.e., 12 h) test [53] at 1000 °C was performed in 2008 with Type II FBGs made in pristine and H_2_-loaded SMF-28 using the phase mask technique and 800 nm, 120 fs pulses. In this time frame, gratings fabricated in pristine SMF-28 experienced a ~300 pm red drift while the gratings made in H_2_-loaded SMF-28 exhibited a much more modest red drift of ~100 pm (see Figure 7).

**Figure 8:**
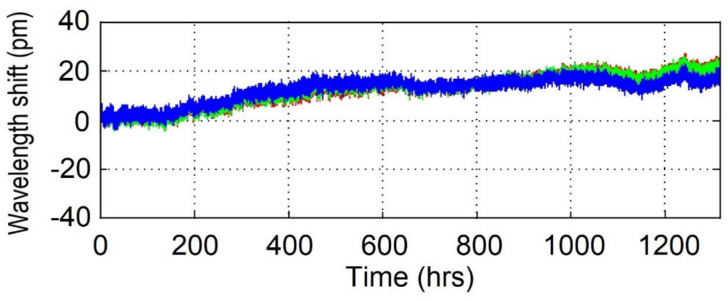
Bragg wavelength drift of 3 Type II FBGs at 650 °C over 1300 h (reproduced from [54]).

A very simple wavelength drift experiment [54] was designed and performed at 650 °C, i.e., the low end of the high temperature range. The rationale behind the test was not to study the wavelength drift behavior at an extreme temperature but rather to establish if there is a high temperature range where fiber sensors can fulfill the strict requirements of thermocouple sensors while maintaining their advantageous characteristics like immunity to electromagnetic field, small size and multiplexing capability. With that purpose in mind, the authors have given great consideration to the accuracy of the results. In order to monitor the furnace temperature, a high-accuracy platinum resistance thermometer (worst case uncertainty of 0.07 °C) with traceable calibration was used to confirm the accuracy of the furnace thermocouple. The Type II FBGs were monitored using a tunable laser interrogator with an internal traceable gas cell specified with a long-term stability of 1 pm. The fibers were placed loosely in quartz tubes to avoid any strain during the process. During the 1300-h test the Bragg wavelength drifted toward longer wavelengths by 20 pm, which would correspond to a positive 0.015 pm/h drift rate (see Figure 8). At 650 °C this wavelength drift rate will correspond to a very low 0.01 °C/h error.

**Figure 9:**
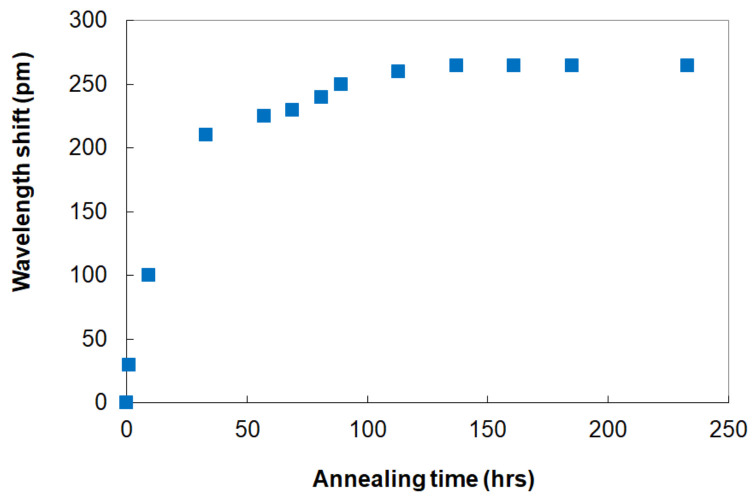
Bragg wavelength drift of π-shifted Type II FBG at 1000 °C (reproduced from [55].

In another experiment [55], an 80 fs Ti-sapphire regenerative amplifier operating at a central wavelength of *λ* = 800 nm was used to inscribe π-shifted Type II FBGs in SMF-28 using a π-shifted phase mask with a period of 3.21 μm. To test how the FBGs responded to high temperature annealing, a number of devices were temperature-cycled many times inside a tube furnace from 20 °C to 1000 °C in ambient air. During this testing (Figure 9), the FBGs remained in the furnace at 1000 °C for more than 200 h. The the π-passband features in the spectra of all the FBGs tested at 1000 °C drifted toward longer wavelengths by 150–200 pm with an average drift rate being ~4 pm/h during the first 50 h of annealing, ~1 pm/h during the following 80 h and <0.1 pm/h during the remaining hours of the experiment. 

**Figure 10:**
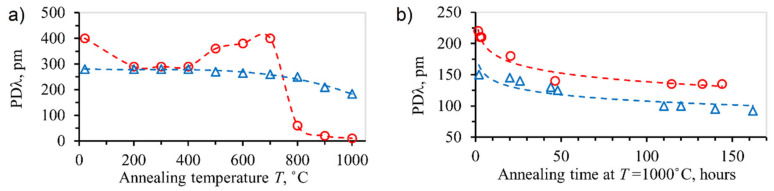
(**a**) Birefringence (PDλ) of π-shifted Type II FBG in SMF-28 (blue triangles) and polarization-maintaining Fibercore HB1500 PM fiber (red circles). (**b**) wavelength drift of π-shifted Type II FBG in SMF-28 for different levels of the initial birefringence (blue: ~2.6 × 10^−4^; red: ~4.2 × 10^−4^), reproduced from [56].

The same Ti-sapphire regenerative amplifier and phase mask were also used to inscribe highly birefringent π-shifted FBGs in SMF-28 [56]. The birefringence (polarization-dependent Bragg wavelength; PDλ in Figure 10) was maximized when the linear polarization of the laser beam was aligned perpendicular to the fiber. The birefringence can grow to ~5 × 10^−4^, which corresponds to a 500 pm polarization dependent drift of the π-phase-shifted passband feature. The birefringence can also be tuned using the ability to erase old nanostructures and simultaneously write new ones by changing the polarization of the femtosecond pulses. When exposed to 1000 °C, the FBG birefringence drops to ~50% of its initial value at room temperature in the first 10–15 min, but then only slowly decreases in the next 100 h of annealing (Figure 10b).

These results refer to the relative difference of the Bragg wavelengths corresponding to the two polarizations and not to the individual wavelengths which should be characterized by the same behavior as presented in [55]. The decay of the birefringence at 1000 °C (Figure 10b) is probably due to the decrease in viscosity of the glass substrate.

**Figure 11:**
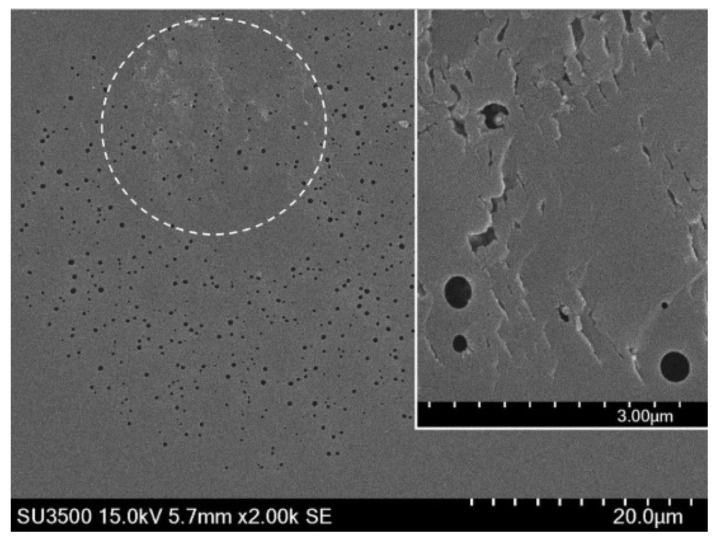
RALF structure and nanogratings inside the FBG in the insert (reproduced from [57]).

In 2018, the earlier mentioned 80 fs Ti:sapphire regenerative amplifier operating at 800 nm and the 3.21 μm–pitch phase mask were used to inscribe uniform Type II FBGs [57] into a pure silica nano-engineered fiber [58] (so called random air-line fiber (RALF)) as well as into SMF-28. Figure 11 [57] shows an SEM image of the RALF’s structure with a magnified image (insert) of a nanograting structure formed inside the Bragg grating planes.

**Figure 12:**
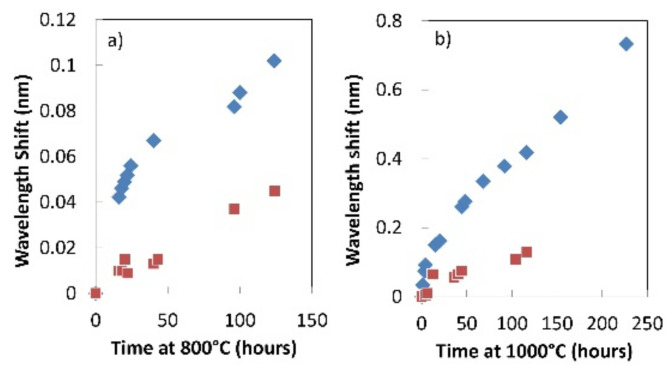
Bragg wavelength drift of Type II FBGs in SMF-28 (red) and RALF (blue) at 800 °C (**a**) and 1000 °C (**b**) (reproduced from [57]).

In order to test the resilience of these FBGs under extreme conditions, a pair of FBGs in RALF and SMF-28 was isochronally annealed at 800 °C for 125 h (Figure 12a). A linear drift of ~0.3 pm/h was measured for the device in SMF-28 and a nonlinear drift rate for the device in RALF of 2.3 pm/h during the first 20 h and 0.45 pm/h for the remainder of this test. At 1000 °C, the FBGs red-drifted at larger rates: ~1.2 pm/h and ~3.5 pm/h for the devices in SMF-28 and RALF, respectively (Figure 12b). It is to be noted that in this experiment the wavelength drift is significantly smaller for SMF-28 compared to RALF, even though there is inherently no dopant diffusion for the latter. No erasure of the index modulation was observed for either grating in this case at 800 °C or 1000 °C.

### 3.4. Experiments Related to Stress Effects in Optical Fibers

**Figure 13:**
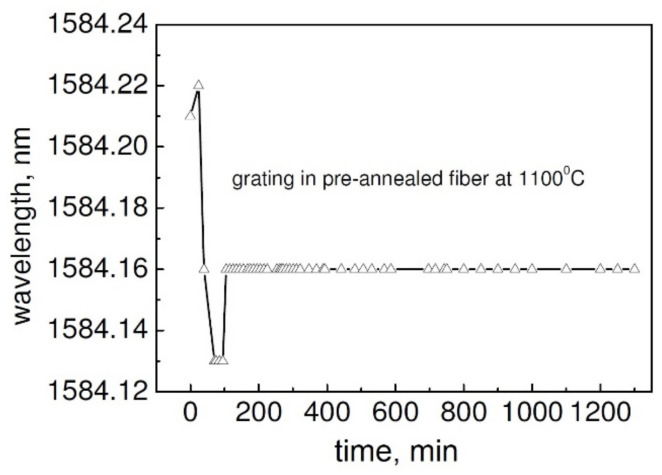
Bragg wavelength drift of a Type II FBG in pre-annealed fiber (reproduced from [59]).

To extend the stability of the FBG structure above 1100 °C, using 800 nm, 120 fs pulses, in 2009 Type II FBGs were inscribed in strands of SMF-28 that were first annealed at 1100 °C for 5 h [59]. The annealing was intended to release the stress in the fiber caused by the fiber manufacturing process. A number of Bragg gratings were also inscribed in the pristine SMF-28. Both sets of the resultant FBGs were then annealed at 1200 °C for 20 h. The FBGs inscribed in pristine SMF-28 completely annealed out at 1200 °C in ~7 h. The FBGs inscribed in SMF-28 that was pre-annealed at 1100 °C lost only ~10% of the initial reflectivity and showed almost no wavelength drift (within the accuracy of the measurement system based on a 20 pm resolution OSA), as can be seen in Figure 13. Repeated temperature cycling performed on the pre-annealed FBGs did not reveal any hysteresis effects. While initial high temperature pre-annealing of the fiber is not presently very practical for subsequent grating inscription, the result seems to indicate that the main cause of the wavelength drift is the pre-existent stress in the pristine fiber rather than the dopant diffusion or the stress induced by the inscription process itself.

**Figure 14:**
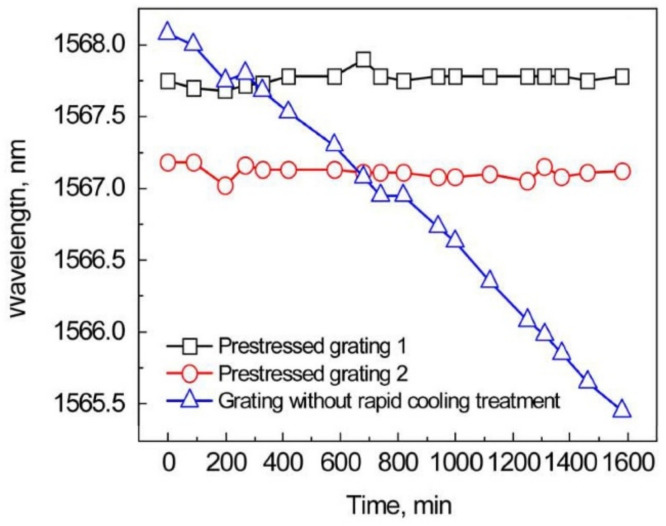
Bragg wavelength drift of prestressed Type II FBGs (reproduced from [60]).

A quenching experiment was performed with a set of Type II FBGs written in SMF-28 using the phase mask technique and a femtosecond laser [60]. Two FBGs were annealed at 1000 °C for 10 h in a tube furnace. During the test the Bragg wavelengths drifted toward the longer wavelengths by ~100 pm for each of the FBGs. After annealing, one FBG was left to cool down in open air, while the other was immersed into liquid nitrogen (quenching) and blue drifts of ~220 pm and ~300 pm were measured for the two FBGs. The authors concluded that different compressive stresses were induced in the fibers. The gratings were then tested again in the furnace at 1200 °C for 26 h. As shown in Figure 14, the Bragg wavelengths of the two FBGs are fairly constant with a very little wavelength drift (a 10 pm resolution OSA was used for the wavelength measurement). In contrast, a similar FBG inscribed in an unprocessed fiber has completely annealed out at this temperature. The authors believe that these results are due to the fact that the compressive stress induced by quenching is compensating the stress induced by heating the gratings at very high temperature.

**Figure 15:**
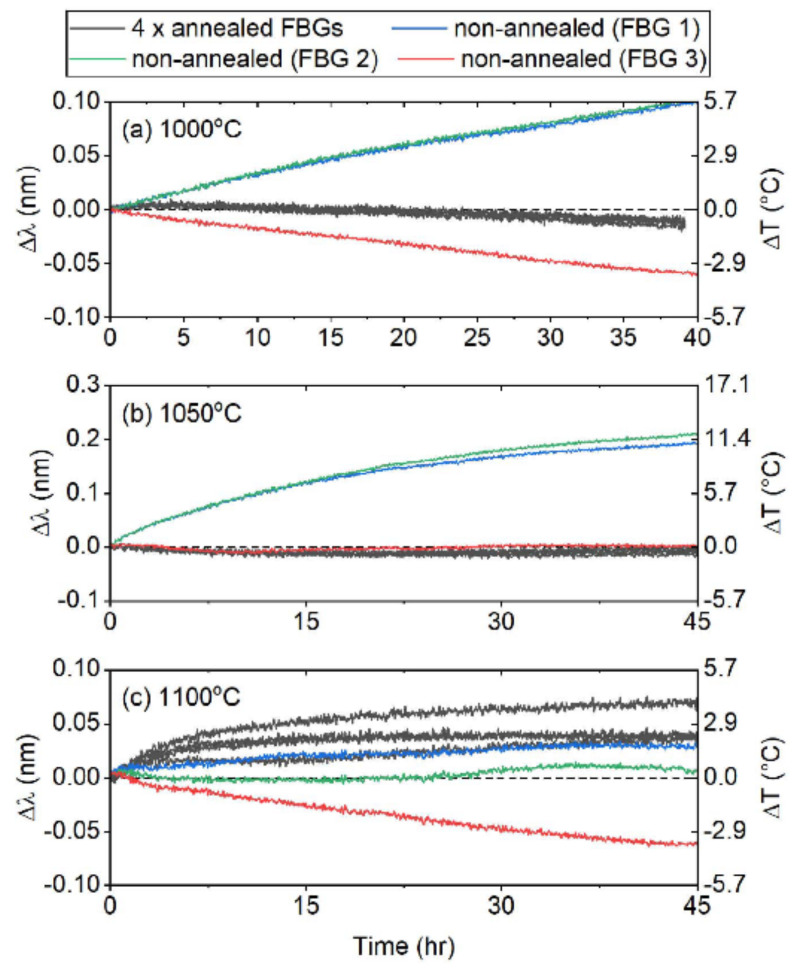
Wavelength drift of SCF gratings at 1000 °C, 1050 °C and 1100 °C (reproduced from [61]).

In 2018 a comparative study was performed on four types of gratings [61], namely: (i) RFBGs, (ii) PbP FBG, (iii) chiral microstructure optical fiber (CDF) [62] and (iv) femtosecond ablation grating made in pure silica suspended core microstructured optical fiber called by the authors SCF grating [63]. The latter two FBG structures were fabricated in pure silica fibers. The first set of reported comparative tests showed the SCF gratings as being the most stable. In the second set of experimental results, four SCF gratings were annealed two times for 4 h at 1125 °C, cooled down to 1000 °C at a rate of 1 °C/h and afterwards to room temperature. These gratings were tested for 48 h together with three other pristine SCF gratings at 1000 °C, 1050 °C and 1100 °C. As presented in Figure 15 [61] at 1000 °C and 1050 °C, the SCF annealed samples showed almost no drift while two non-annealed samples were affected by red and blue drift of ~4 pm/h and 2 pm/h rate, respectively. At 1100 °C all but one of the FBGs drifted. The results of this second set of experiments seem to support the idea of greater wavelength stability at a certain temperature achieved by the higher temperature annealing. The general conclusion drawn by the authors is that the main cause of wavelength drift for doped fiber up to 1000 °C is the modification of the core effective refractive index because of the diffusion of the dopants, while above 1000 °C the wavelength drift may be due to stresses that were formed during grating inscription, fiber drawing and devitrification of silica. Theoretically, the crystallization should be very small at 1100 °C [64], however surface contaminants can dramatically increase crystallisation and protective packaging is required.

### 3.5. Experiments Related to Dopant Diffusion Effects in Optical Fibers

The wavelength drift at high temperatures could be caused by different factors including the diffusion of dopant in the core of the fiber. Such a structure can be created by twisting a PCF locally in such a way as to create a periodic structure. In [62] a helical variation of the refractive index was achieved by twisting a PCF fiber locally inside a micro-furnace resulting in a chiral diffraction grating that was annealed at 900 °C for over 1400 h with almost no wavelength drift (less than 10 pm) over that time period. Before the start of the long-term test, the grating was annealed at 1120 °C for 10 h and then cooled down to room temperature. Since there is no dopant in the core, the results could be interpreted as supporting the idea that it is dopant diffusion that generates the wavelength drift but does not eliminate the stress relaxation as a cause of the wavelength drift.

The core dopant diffusion at high temperature was evaluated using a Fabry-Pérot (F-P) cavity made of a 60 µm section of SMF-28 with its end faces coated with a low reflectivity Cr-layer [65]. The optical phase change of the F-P cavity in SMF-28 was compared to the optical phase change of a 55 µm long F-P cavity made of pure silica PCF. Both cavities were placed in a furnace at 900 °C for ~200 h, then at 950 °C for 120 h, then at 1000 °C for 75 h and, finally, at 1050 °C for 120 h. For better visualization, the phase shifts accumulated during the different multi-hour stages of annealing were converted into fictitious short term temperature variations that would cause phase drifts of the same magnitude [65]. Accumulated optical phase shifts corresponding to a 4 °C, 2.5 °C, 9 °C and 18.1 °C temperature variation were detected in the F-P cavity in SMF-28 respectively <1 °C, 2 °C, <1 °C and 11.4 °C at 900 °C, 950 °C, 1000 °C and 1050 °C. The fact that up to 1000 °C there was almost no change in the PCF cavity, but significant changes occurred in the SMF-28 cavity point to the possibility of dopant (Ge) diffusion due to the high temperature. The drift of both cavities at 1050 °C indicates that other processes are also involved.

**Figure 16:**
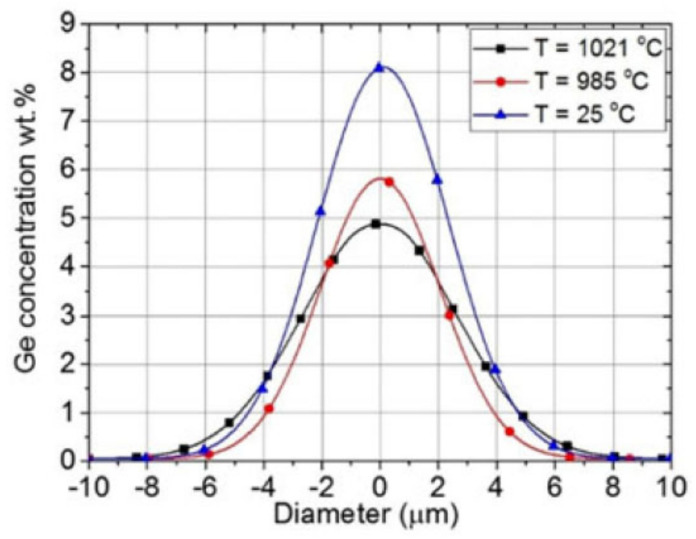
Ge-concentration in the fiber core as a function of position and temperature (reproduced from [65]).

In order to directly analyze the magnitude of dopant diffusion, the radial distribution of the Ge-dopant after the annealing process was measured using Energy Dispersive X-ray analysis (SEM/EDX) on polished end surfaces of the fiber samples subjected to different temperatures. Figure 16 [65] presents Ge-distributions at room temperature, 985 °C and 1021 °C. Of note, at 1021 °C the core diameter has increased from the original value of ~8 μm to ~10 μm.

### 3.6. Influence of Glass Chemical Composition on Silica Glass Stability at High Temperature

Another parameter that can affect the wavelength drift of FBGs at high temperature is the chemical composition of the host fiber. Commercially available silicate fibers include a pure silica core fiber that usually has a fluoride-doped cladding, silica fiber with low and high germanium concentration in the fiber core, fiber whose core is co-doped with boron and active fiber. There are no published comprehensive studies that would correlate the stability of the Type II FBGs with the chemical composition of the fibers, but individual studies have shown that pure silica core fibers and fibers with low germanium concentration are more stable at high temperature than fibers with high germanium concentration. A thorough study is very difficult and expensive to conduct as it would require customized fabrication of numerous samples of special fiber.

**Figure 17:**
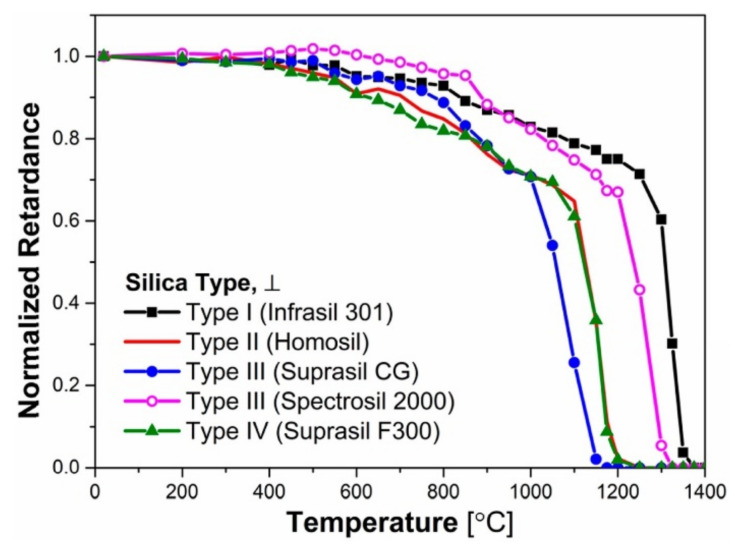
Influence of impurities on glass stability, as deduced by measuring the retardance of embedded nanogratings (reproduced from [66]).

An alternative approach was used in [66] where the authors performed a detailed study of Type II refractive index changes in bulk silica induced by 1030 nm, 250 fs laser pulses. The resultant Type II modification is based on birefringent nanograting structures in glass and its stability at high temperature was evaluated by measuring the optical retardance, using a polarizing optical microscope equipped with a compensator. The irradiated samples were isochronally heated with a temperature step of 50 °C for 30 min up to 1400 °C. After each annealing step the samples were brought to room temperature and the retardance was measured and normalised to the value obtained on the pristine samples. Three experiments were performed to investigate the influence of glass chemical composition on the stability of the nanograting structure. The first study investigated the influence of impurities such as Cl and OH in the glass; the second, the behavior of GeO_2_-SiO_2_ binary glasses and the final study focused on the effect of other dopants like TiO_2_- and B_2_O_3_. According to the results presented in Figure 17, high levels of OH correlate with a lower degree of thermal stability. The nanogratings made in Infrasill 301 (low content of OH) were quasi-stable up to 1300 °C while the nanogratings made in Suprasil GC started disintegrating at 1000 °C.

**Figure 18:**
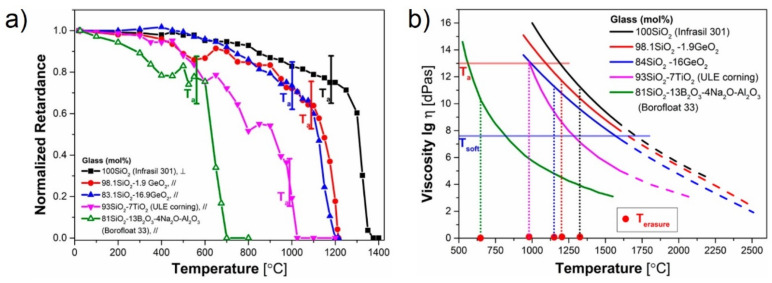
Influence of doping on (**a**) the glass stability, as deduced by measuring the retardance of nanogratings, and (**b**) the glass viscosity with temperature (reproduced from [66]).

Figure 18 [66] presents the behavior of nanogratings in four glasses with different concentrations of GeO_2_. There are only small differences between the four glasses, which the authors attributed to the fact that GeO_2_ and SiO_2_ form a relatively continuous mixture whose viscosity and, therefore, thermal stability are not changed much until a certain concentration of GeO_2_ is exceeded. Doping silicate glasses with other oxides (TiO_2_ or (B_2_O_3_) significantly affects the high temperature thermal behavior (Figure 18a) to the extent that the erasure temperature of nanogratings for B_2_O_3_ drops to almost 700 °C. The measured viscosity of the same glasses with temperature presented in Figure 18b is correlated well with the retardance measurements.

A conclusion was drawn that nanogratings in pure silica glass can survive above 1300 °C, while any doping of the glass deteriorates the nanograting structure at 1000 °C or even below by decreasing the glass viscosity.

### 3.7. High Temperature Effects on Light Scattering in Optical Fibers

**Figure 19:**
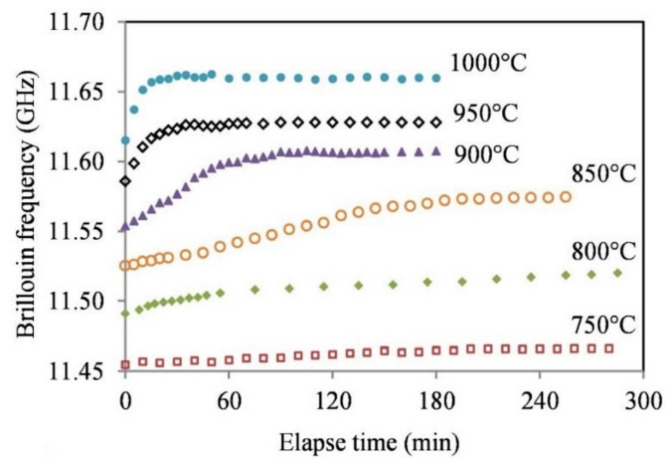
Isothermal Brillouin frequency drift (Reprinted with permission from [67] © The Optical Society [67]).

In 2016, a single mode fiber with a 8.2-μm-diameter core and a 125-μm-diameter cladding was exposed to temperature in the range from 750 °C to 1000 °C for multiple hours [67]. The temperature measurements were performed using the PPP-BOTDA technique [68], which is based on measuring the Brillouin frequency of stimulated Brillouin scattering. There is a drift of Brillouin frequency that will decrease to zero after a period of time that depends on the temperature, with the effect being more pronounced at higher temperatures (see Figure 19). The magnitude of the observed temperature-induced shift of the Brillouin frequency at 1000 °C (~50 °C) would correspond to an ~850 pm Bragg wavelength drift of an FBG. This would be a very large drift which is rarely observed in FBG experiments. Since the diffusion of the dopant (Ge) will lead to a decrease in the refractive index and therefore a decrease in the Brillouin frequency, the authors consider the dopant dilution to be a minor effect, the main cause for the Brillouin frequency drift being the release of stress.

### 3.8. Summary of the FBG Wavelenght Drift at High Temperature

A careful analysis of the literature results presented in Section 3.1, Section 3.2, Section 3.3, Section 3.4, Section 3.5, Section 3.6 and Section 3.7 shows that there is a general trend observed in all annealing experiments. The FBGs presented in [47,48,49,51,53,55,57] were all affected by a strong red wavelength drift in the first 100 h and the FBGs in [47,48,49,51] which were tested for longer than 100 h showed a slow blue wavelength drift after an initial blue drift. While, according to Figure 3, the red-blue drift pattern continued for the whole duration of the 9000-h experiment, for annealing temperatures close to 800 °C, at 900 °C the drift pattern became more complicated after 3000 h. This observation is confirmed by the results presented in Figure 4 that show that at 950 °C the red-blue drift pattern ended even earlier, after about 2000 h of annealing. The drift pattern presented in Figure 2 does not fit the other results. The absence of the red-blue pattern in Figure 6b could be the consequence of inscribing the FBGs with picosecond pulses which tend to induce very strong modification in glasses.

On the other hand, the tests dealing with potential causes of the wavelength drift (Section 3.4, Section 3.5 and Section 3.6), do not convey a clear message as, for instance, [63,65,67] claim opposite effects of dopant diffusion. The experimental results in [65] may be affected by stresses induced in the cavity during the heating of the structure or even changes in the cavity length. It is however obvious from the results presented in [59,60,63] that stress in the fiber is a major, if not the main cause of the wavelength drift. The behavior of optical birefringence/retardance (which is caused by the presence of light-induced nanogratings) at high temperature (Figure 17 and Figure 18) shows the potential of silica fiber to withstand temperatures up to 1300 °C, but also the effect of dopants and impurities to drastically reduce the high temperature limit. While, strictly speaking, these results at extreme temperatures were not obtained during isothermal annealing, the results of isothermal annealing at 1000 °C (Figure 10b) show the same trend, i.e., a strong decay of birefringence.

## 4. Materials and Methods

For our experiments, a large number of FBGs were fabricated in SMF-28 using the phase mask technique and femtosecond laser pulses, i.e., the FBGs under consideration are Type II FBGs following the terminology introduced earlier. It is well known (see Literature Review Section 3 that FBGs at a high constant temperature experience a Bragg wavelength drift which can range from a few picometers to hundreds of picometers depending on the annealing time. In order to allow measurements of spectral drifts with a picometer precision, all the prepared Type II FBGs were in fact π-shifted Type II FBGs, i.e., FBGs that have a very narrow pass-band feature in the center of their resonance spectra. The π-shifted Type II FBGs were fabricated using the inscription procedure presented in [55]. Very briefly, the 80 fs pulses (Fourier transform-limited pulses) and 400 fs pulses (chirped pulses) generated by a Ti-sapphire regenerative amplifier operating at *λ* = 800 nm were used for the inscription.

**Figure 20:**
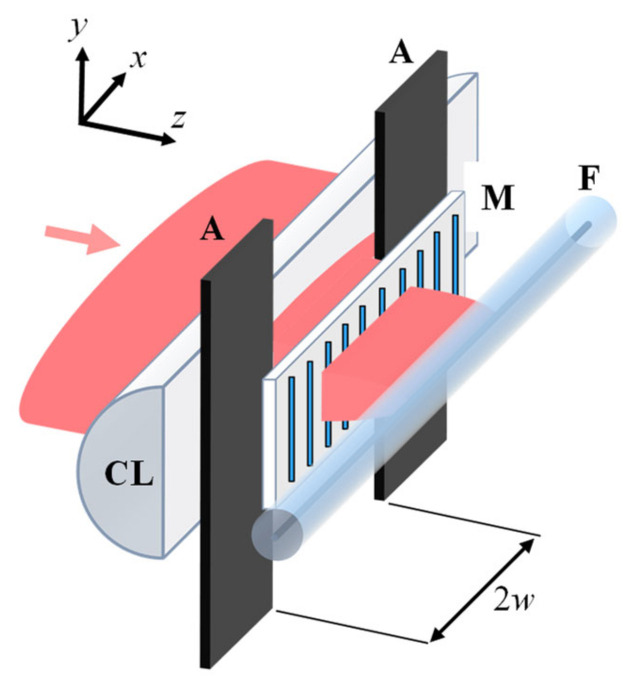
Schematic of the setup for inscription of π-phase-shifted Type II FBG.

**Figure 21:**
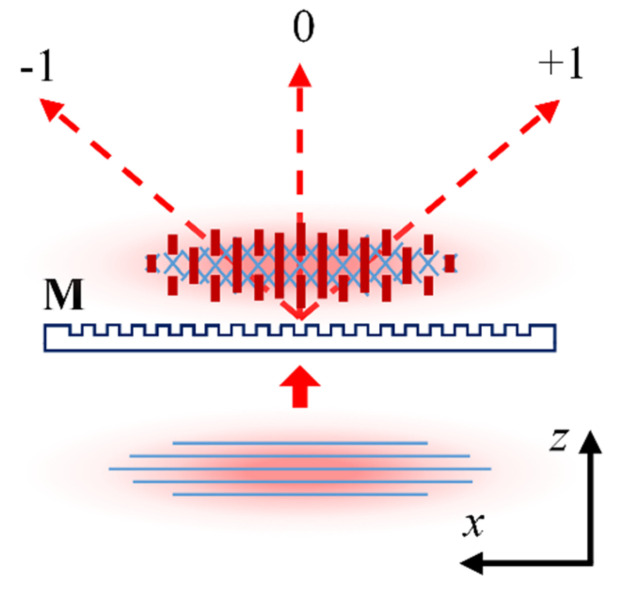
Multiple beam interference pattern produced near a first order phase mask.

The output Gaussian beam ~7 mm in diameter at the 1/*e*^2^ intensity level was expanded ~3 times along the *x*-axis and focused through a phase mask (M) using a cylindrical lens (CL) with a focal distance of 15 mm (see Figure 20). A 7 mm-wide vertical slit aperture (A) was used to select the central part of the beam. Two phase masks with a pitch Λ**_1_ = 1.07 µm (first-order mask) and Λ**_3_ = 3.21 µm (third-order mask) were used to fabricate the FBG samples. Depending on the distance between the optical fiber (F) and the phase mask (M), the interference pattern at the fiber could be produced by the beams from mainly +1/−1 diffraction orders or the beams originating from all of the available diffraction orders, i.e., 0 and +1/−1 diffraction orders in the case of a first-order mask and 0, +1/−1, +2/−2 and +3/−3 diffraction orders in the case of a third order mask [69]. An example of a multiple beam interference pattern (0 and +1/−1 diffraction orders) produced by a first order mask at a small fiber-to-mask distance is schematically shown in Figure 21.

During inscription the π-shifted Type II FBGs were continuously monitored with a tunable laser system and the laser exposure was stopped when their strength reached approximately −15 dB in transmission. The prepared FBG samples were then spliced to fiber pigtails terminated with APC connectors and placed into a Lindberg Blue M Tube Furnace (Lindberg/MPH, Riverside, MI, USA) for annealing. Three identical Lindberg furnaces were used, each accommodating 4–5 samples. An external Type K thermocouple (Omega, St-Eustache, QC, Canada) was placed in each furnace, in close proximity of the furnace’s built-in thermocouple, and monitored using data acquisition software. FBGs were centered within the furnaces, in-between the two thermocouples (external and internal), loosely resting in a hollow fused silica semi-cylinder so that they were free from strain and not touching the furnace side walls. Using FBG optical interrogators, each FBG was monitored independently using two separate interrogator channels, with one channel monitoring reflection and the other monitoring the light transmitted through the FBG and an optical isolator.

Full spectrum optical data was generally collected every two hours, with the Bragg peak wavelengths being logged every 5 s in order to facilitate data processing and provide additional resolution in time. Two interrogators were used: a Hyperion SI255 FBG Interrogator (Micron Optics, Atlanta, GA, USA) with 16 Channels and 8 pm wavelength resolution and a Micron Optics SM125 FBG Interrogator with four Channels and 5 pm wavelength resolution.

In a separate experiment, three π-phase shifted gratings, prepared under identical conditions to the ones above, were then packaged in a protective quartz tubing. Their long-term wavelength stability was studied in 0–1000 °C temperature range with 0.1 pm wavelength resolution using an 8164 Lightwave Measurement System (Agilent, Santa Clara, CA, USA) with an integrated tunable laser and detector modules. For the packaged fibers, only the wavelength drifts measured at 1000 °C in the horizontal tube furnace are reported in this paper, while the bulk of the results will be reported elsewhere. The temperature of the annealing furnace was controlled with an external S-type thermocouple. The tips of the packaged fibers with inscribed FBGs were placed in a massive Inconel block (to decrease temperature fluctuations below 0.1 °C) and placed in the middle of the furnace where the temperature profile of the furnace has a maximum. The drift rate at 1000 °C in pm/h was calculated by dividing the Bragg wavelength changes at 600 °C, which were measured in the caesium pressure-controlled heat pipe [70] before and after the annealing at 1000 °C, by the known annealing time at 1000.

## 5. Experimental Results

Five independent annealing tests were run at 900 °C and 1000 °C. In each test, a set of either 4 or 5 Type II FBGs fabricated under the same experimental conditions and with similar spectral characteristics was annealed for a time duration of up to 1000 h. The purpose of the tests was to gather information regarding the influence of the laser writing conditions, annealing temperature and annealing history on the drift of the wavelength of the π-phase shift passband feature. The wavelength of the passband feature in the FBG spectrum will be called the ‘Bragg wavelength’ to avoid confusion. Three different types of gratings were tested, each of them being a Type II FBG made with different exposure condition and resulting in a different grating structure as presented above in Section 3.3, namely:

(1) Type-a gratings made with 80 fs pulses (Fourier-transform-limited pulses) and a first-order phase mask;
(2) Type-b gratings made with 400 fs pulses (chirped pulses) and a third-order phase mask;
(3) Type-c gratings made with 80 fs pulses (Fourier-transform-limited pulses) and a third-order phase mask.

Type-a, Type-b and Type-c FBGs were inscribed using a 0.7 mJ, 0.96 mJ and 1.03 mJ pulse energy, respectively. This choice of pulse energy was made in order to equalise the growth rate of the different types of FBGs. Type-a, Type-b and Type-c FBGs did not show any statistical difference between their spectra. A typical spectral shape of the pi-phase shifted FBGs fabricated for the present experiment is presented in Figure 7b of [55], with the −3dB bandwidth of the π feature being in the 30–40 pm range.

The wavelength of the π-phase shifted passband feature in the FBG spectrum was recorded every two seconds to measure the spectral drift, while the FBG spectra were recorded every two hours to evaluate the transmission loss as a function of time.

### 5.1. Annealing of Type-a FBGs at 1000 °C Followed by Annealing at 900 °C

**Figure 22:**
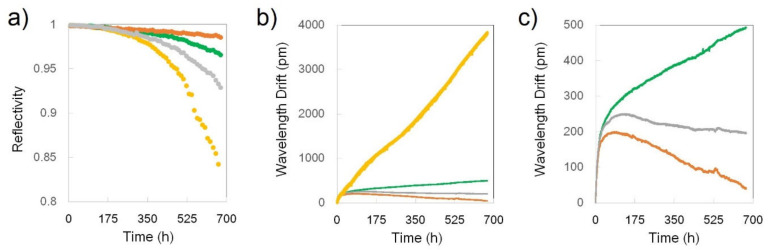
Changes to reflectivity and wavelength of 4 Type-a FBGs during annealing at 1000 °C. (**a**) variation in reflectivity with annealing time, (**b**) wavelength drift of Bragg resonance, (**c**) zoom-in on the 3 slowly-drifting Type-a FBGs.

Four FBGs of Type-a were placed in the furnace at 1000 °C and left at this temperature for 660 h. The FBGs were continouslly monitored both in transmission and reflection. Variations in grating reflectivity are shown in Figure 22a. Figure 22b displays the corresponding drift in the grating Bragg resonance while Figure 22c is an expanded view of the 3 slow drift gratings in the experiment.

As can be seen in Figure 22b) the set of four Type-a FBGs is split in two categories: a very fast wavelength drift grating with a 5 pm/h drift rate and a set of 3 low drifting rates gratings (0.4, 0.25 and 0.15 pm/h) presented also in Figure 22c. The wavelength drift values presented above are only referring to the time range when the drift rate is quasi-constant, generally after the first 100 h of annealing. In the first annealing stage, especially in the first 5–10 h, all the FBGs have extremely high drift rates, above 20 pm/h, which is also true of the majority of the published results so far. The fast-drifting FBG (yellow trace) is obviously an outlier as its drift rate of 5 pm/h is more than 30 times faster than that of the FBG with the slowest drift rate (grey trace; 0.15 pm/h). However, there is also variability among the slowly drifting FBGs in terms of the sign of the drift. The characteristic wavelengths of two FBGs (grey and orange traces) move toward shorter wavelengths (blue drift), whereas the characteristic wavelength of one FBG (green trace) is moving toward longer wavelengths (red drift). However, there is no obvious correlation between the loss in the refractive index modulation (reflectivity) and the changes in the local effective refractive index (wavelength drift), except for one FBG (yellow trace in Figure 22a).

**Figure 23:**
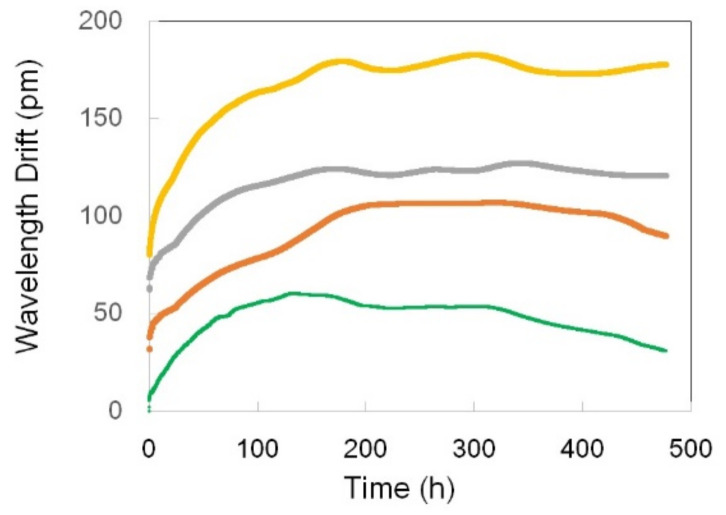
Wavelength drift at 900 °C of the Type-a FBGs that were pre-annealed at 1000 °C.

In the second part of this test, the temperature was decreased from 1000 °C to 900 °C in ~1 h and left at this temperature for ~500 h. For the entire second part of this test, the reflectivity of the FBGs remained constant, a proof that the refractive index modulation remained unchanged. There was however wavelength drift in the first 150 h and, as before, especially in the first 50 h (drift rate from 0.4 pm/h to 1 pm/h). For the remainder of the test, the FBGs were remarkably stable (less than 0.1pm/h drift rate), with only one FBG (green trace) showing a slight blue drift (Figure 23).

### 5.2. Annealing of Type-b FBGs at 1000 °C Followed by Annealing at 900 °C

**Figure 24:**
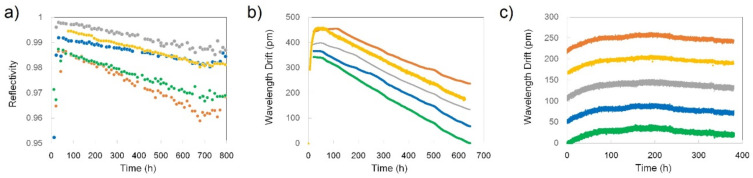
(**a**) Change in the reflectivity of Type-b FBGs at 1000 °C (from 0 h to 700 h) and at 900 °C (from 700 h to 800 h); (**b**) Wavelength drift of Type-b FBGs at 1000 °C; (**c**) Wavelength drift at 900 °C of the Type-b FBGs that were pre-annealed at 1000 °C shown in (**b**).

The behavior of the five Type-b FBGs in this set is quite different from that of the Type-a FBGs. First, the grating structure of Type-b FBGs is much more stable than that of Type-a FBGs. Indeed, the reflectivity of Type b FBGs decreased only by a couple percent during annealing at 1000 °C for ~700 h (Figure 24a). Second, the characteristic wavelength of Type b FBGs during the 1000 °C annealing period behaved more consistently than in the case of Type a FBGs. After the first few hours of fast drift, all the gratings experienced 50–100 h of stable wavelength followed by a blue drift with the drift rate in a very narrow range. Four of the five FBGs have the wavelength drift rate in the range between 0.31 pm/h and 0.48 pm/h (Figure 24b). When the temperature was decreased to 900 °C at the end of the 700-h run at 1000 °C, the FBGs’ reflectivity completely stabilized (Figure 24a), whereas the wavelength drift was still present. A relatively fast red drift at a ~0.5 pm/h rate in the first 75 h was followed by a period of wavelength stabilization and then by a much slower blue drift at a ~0.05 pm/h rate (Figure 24c). To be noticed also is the high degree of similarity between all the traces in Figure 24.

### 5.3. Annealing of Type-c FBGs at 1000 °C Followed by Annealing at 900 °C

**Figure 25:**
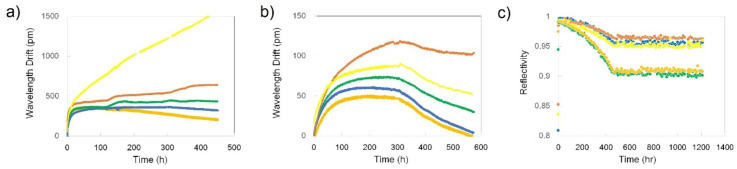
(**a**) Wavelength drift of Type-c FBGs at 1000 °C, (**b**) Wavelength drift at 900 °C of the Type-c FBGs that were pre-annealed at 1000 °C and shown in (**a**), (**c**) Type-c FBGs reflectivity change during annealing at 1000 °C and 900 °C.

Five Type-c FBGs were tested at 1000 °C for ~500 h (see Figure 25a). There was a rapid non-linear red drift at the beginning of the test of more than 10 pm/h rate on the average. After ~20 h, the wavelength drift became linear, with four FBGs having drift rates in the range ±0.25 pm/h. As in the case of the Type-a FBGs, there was an outlier (yellow trace) with a significantly faster drift rate (i.e., 3 pm/h). The sign of the drifts also varied: two FBGs had a red drift and three had a blue drift.

Following the annealing protocols for Type-a and Type-b FBGs, the temperature was decreased to 900 °C (Figure 25b). After experiencing a ~1pm/h red drift rate during the first 50 h at 900 °C, the wavelength of 4 FBGs stabilized almost completely. However, after the 300-h mark all the gratings started drifting toward blue wavelengths with rates from 0.02 pm/h to 0.1 pm/h.

As can be seen in Figure 25c, there is a decay of Type-c grating structure similar in amplitude to the one undergone by Type-a FBGs (Figure 22a). During almost 500 h of annealing Type-c FBGs lost between 5% and 10% of their reflectivity (Figure 25c). However, the reflectivity almost completely stabilised at 900 °C.

### 5.4. Annealing of Type-b FBGs at 900 °C Without Pre-Annealing

**Figure 26:**
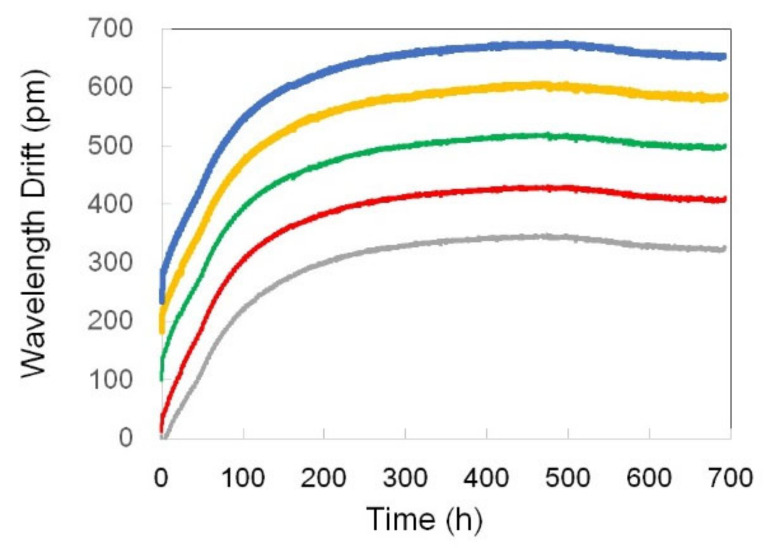
Wavelength drift of Type-b FBGs at 900 °C without pre-annealing at 1000 °C.

For this experiment, a set of 5 Type-b FBGs was tested directly at 900 °C without pre-annealing at 1000 °C in order to compare the annealing behavior at this temperature with that of the Type-b FBGs that were first pre-annealed at 1000 °C for hundreds of hours and then monitored at 900 °C. The drift toward longer wavelengths (Figure 26) at the beginning of the annealing test (2–3 pm/h drift rate) was much higher than the one (0.5 pm/h) measured for the Type-b FBGs pre-annealed at 1000 °C (Figure 24c). It can also be seen that the total red drift (~300 pm) was much larger than in the case of the pre-annealed Type-b FBGs (~20 pm).

### 5.5. Annealing Packaged Type-c FBGs at 1000 °C

**Figure 27:**
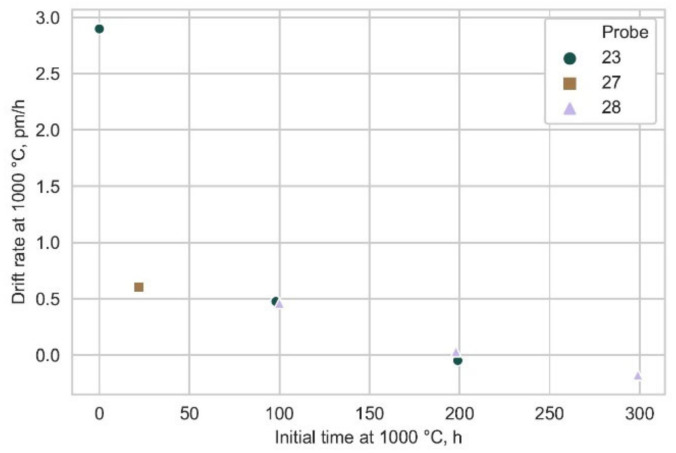
Drift rate at 1000 °C for 3 Type-c FBGs packaged in quartz tubing, calculated by comparing the wavelength at 600 °C before and after annealing at 1000 °C.

Three Type-c FBGs were packaged in quartz tubing and the drift rates were measured in the 0–1000 °C temperature region to assess the influence of packaging on FBG performance. Only the drift rates obtained at 1000 °C in the horizontal tube furnace are shown in this paper in Figure 27 for the purpose of the comparison.

As explained in detail in Section 4, the drift rate of packaged fibers at 1000 °C was calculated post-factum, so the presentation of Figure 27 differs from Figure 25. Nevertheless, the wavelength drift pattern of packaged fibers remains essentially unchanged. We notice that after 100 h the drift rate drops below +0.5 pm/h and this value continues to decrease with annealing time at somewhat slower pace after the 100 h mark. Interestingly, after 300 h at 1000 °C for probe 28 we registered a negative drift rate of 0.18 pm/h (blue shift). This signifies that chosen packaging does not alter the wavelength drifting behavior of FBGs dramatically–a welcome development for applications outside the research laboratory.

### 5.6. Summary of the Experimental Results

The wavelength drift pattern presented as a general feature in the Literature Review section also holds true for the above results at 900 °C, 1000 °C, or at 900 °C after annealing the FBGs for hundreds of hours at 1000 °C. This pattern is made of a fast wavelength drift at the beginning of the annealing process and a slow blue secondary drift. The red drift rate for annealing at 900 °C and 1000 °C is 1–2 pm/h and 5–20 pm/h, respectively. The blue drift rate is 0.05–0.1 pm/h and 0.2–0.5pm/h for the annealing at 900 °C and 1000 °C, respectively. It is also characteristic to the wavelength drift pattern that in between the periods of red drift and blue drift there is a period of almost no drift for 50–300 h.

While this is a general conclusion reached by analyzing the drift patterns, there is also a certain degree of variability among the results. In each set of Type-a and Type-c FBGs we tested, there was an outlier in terms of drift rate. This indicates that in these cases, and to a certain extent in the case of Type-b FBGs, there is variability in the internal structure of individual FBGs. This variability is most likely caused by the different exposure conditions of the fiber core to the femtosecond radiation. Type-a FBGs were written at a small fiber-to-mask distance (~50 μm) where the beams from 0 and +1/−1 diffraction orders strongly contribute to the resultant Talbot-like interference pattern [13]. The same is also true of Type-c FBGs in which case the fiber was placed ~150 μm away from the mask and all diffraction orders (i.e., 0, +1/−1, +2/−2 and +3/−3 diffraction orders) contributed to the interference pattern. Even if there is a small variability in the fiber-to-mask distance (e.g., ~2 μm) during inscription, the fiber core will be exposed to essentially different interference patterns and, as a result, prone to high structural variability. On the other hand, Type-b FBGs were written at a ~500 μm fiber-to-mask distance where the walk-off effect [13] reduces contributions from 0, +2/−2 and +3/−3 diffraction orders to the interference pattern, making it resemble a two-beam interference pattern. This can explain why the drift rates of Type-b FBGs presented in Figure 24b,c and Figure 26 are very similar in shape and have no pronounced outliers.

Beside the variability of drift rates within individual FBG sets, there is also a group variability among the three types of FBGs tested. While Type-b FBGs are quite different in their behavior compared to Type-a and Type-c FBGs, there are only small differences in the behavior of Type-a and Type-c FBGs, which is unexpected as the periods of FBGs made with first- and third order phase masks are obviously different.

The most important results of the annealing tests are (i) the difference in the red drift rate for Type-b FBGs that were annealed directly at 900 °C (2 pm/h; Figure 26) and those that were annealed at 900 °C after pre-annealing at 1000 °C (0.5 pm/h; Figure 24c), (ii) the same blue drift rate for these two scenarios, which indicates that pre-annealing does not have any effect on the blue drift mechanism and (iii) the fact that the same trends are observed with packaged FBGs, which shows that a properly designed package does not affect the long-term behavior of the FBG temperature sensors inside it.

Based on these observations, there is no straightforward answer to the question if the wavelength drift pattern is caused by temperature-induced changes in the refractive index of the optical fiber or re-organization of the grating structure. Nonetheless, the commonality of the red and blue drift patterns for widely different grating structures seems to indicate that the wavelength drift is primarily determined by the high temperature behavior of the optical fiber itself.

## 6. Discussion

The experimental results above, from previous reports and our new experimental studies presented in this paper, show that the accuracy of long-term temperature monitoring using FBGs will be strongly affected by isothermal Bragg wavelength drift if measurements are performed at high temperatures, i.e., at 800–1000 °C. This can be seen by converting the accumulated drift of the Bragg wavelength (i.e., the integral of Bragg wavelength drift over time) into a fictitious short-term temperature variation (i.e., an error in temperature measurements) that would cause a Bragg wavelength drift of the same magnitude. More specifically, to compete with standard high temperature thermocouples in terms of accuracy, the wavelength drift of a FBG should not exceed a few tens of picometers if one assumes that a 16.8 pm change in the Bragg wavelength corresponds to a ~1 °C temperature variation in the 800 °C to 1000 °C temperature range [18]. So far, a measurement error that is comparable in the above context to that of thermocouples, i.e., ~1.5 °C during 1300 h of annealing, could be achieved by high-temperature-resistant FBGs, namely RFBGs, PbP FBGs and Type II FBGs that are not thermally erased during prolonged exposure 1000 °C, only at a rather ‘modest’ temperature of 600 °C [54].

As mentioned above, the experimental data presented in the literature, fall into two distinct categories: the wavelength drift patterns of FBGs during 12 to 9000 h of annealing at 900 °C to 1000 °C and the results of high temperature tests specially designed to study processes that may affect the wavelength drift, such as dopant diffusion, stress and glass chemical composition.

The diverse results on the Bragg wavelength behavior at 1000 °C have nonetheless a certain degree of commonality. The most evident common feature of all the tests presented here is a very fast red drift (2–50 pm/h rate) in the beginning of 900 °C or 1000 °C annealing tests followed by a slower blue drift that can last for hundreds of hours. The red wavelength drift can also last for 100–200 h but the majority of the wavelength drift takes place in the first 10–30 h after the 1000 °C temperature has been reached. This trend is observed in [47,48] at 1000 °C, in [49] at 890 °C, [51] at 920–950 °C, [53,55,57] at 1000 °C, and our annealing tests. This trend is common to different FBGs types, i.e., regardless of the inscription technique/method and characteristics of the laser pulses, which seems to indicate that wavelength drift is primarily associated with the behavior of the fiber substrate at high temperature rather than material modification induced by the laser radiation. The single instances where the red drift was not observed or was small are (i) in [61], where the FBGs were first annealed at 600–800 °C before being annealed at 900 °C and 1000 °C, and (ii) in [59], where the FBGs were pre-annealed at 1100 °C and [60].

What is the cause of the rapid red drift? As it was mentioned earlier in the Introduction, there are several processes that can cause isothermal wavelength drift at high temperature. First, it is dopant diffusion, a well-known effect used in industry to enlarge the core of the fiber in a process called Thermally Expanded Core (TEC) [71]. While TEC is usually performed at 1100–1200 °C, dopant diffusion also occurs at 1000 °C and even lower temperatures [65]. In the case of germanium-dopant present in standard optical fibers, the dopant diffusion at 1000 °C will result in a −0.0025 negative change in the effective refractive index and lead to a ~250 pm blue wavelength drift [72]. While a measurable effect at 1000 °C, the dopant diffusion cannot obviously be the cause of the red drift, as it was earlier mentioned in [67].

Second, it is the fiber manufacturing process. During the drawing of the optical fiber and the fast cooling of the fiber, the internal stresses are frozen into the fiber (frozen-in stress). The residual stress induced during fiber drawing can be very large and thus cause changes in the refractive index of the optical fiber through the photoelastic effect [73,74]. According to theory and experimental results, frozen-in stress (strain) will be released when a fiber is annealed close to its fictive temperature. Using spectral interferometry it was shown that after annealing, the diameter of the fiber decreased by 200 nm resulting in potential refractive index changes in excess of 0.02 % [46,75]. In one case, a very short (a few seconds) annealing at 1400 °C of a short piece of optical fiber resulted in a large change in the refractive index [75].

It is possible that frozen–in stresses are released during annealing at ~1000 °C, causing an increase in the fiber refractive index and, as a consequence, a red drift of the Bragg wavelength at the beginning of the 1000 °C annealing process. High temperature pre-annealing at temperatures in excess of 1000 °C, such as 1100 °C or 1200 °C could release frozen-in stresses and thus mitigate the red drift during the first stage of annealing at a lower temperature [59,60]. This can also explain the compression effect reported in [60].

The second stage of the annealing process at 1000 °C consists of a slow blue drift ranging between 0.1 and 0.5 pm/h that lasts for hundreds of hours. Based on the definition of the Bragg wavelength, Equation (1), the blue wavelength drift should result from either a decrease in the value of the effective refractive index or a reduction of the grating length. As it is hard to believe that the Bragg grating contracts at a high temperature, the blue drift should be mainly correlated with a slow change of the effective refractive index of the fiber substrate and/or the grating. The dopant diffusion could lead to a blue drift but, according to published reports, by the time the blue drift starts, (i.e., after 200–300 h), the diffusion process will have already been completed as it reaches equilibrium in less than 100 h [72]. Moreover, at 1000 °C the frozen-in stresses should have been released after 200–300 h. Devitrification (crystallization) has not yet reported during high temperature annealing of fibers, but if devitrification does take place, the change in refractive index would be positive. All in all, there is no explanation of the blue drift at the moment.

## 7. Conclusions

For FBGs to have a future as temperature sensors in the 900–1000 °C range, the phenomenon of Bragg wavelength drift needs to be addressed. According to the review of published results and our recent experiments presented in this paper, the drift exhibits two types of behavior: a fast red drift in the first 100–200 h and a slow blue drift after a “no-drift” period. As it seems that the red drift is caused by the release of frozen-in stresses generated during the fiber manufacturing process, its negative effect on the temperature measurement can be somewhat mitigated by pre-annealing the FBG sensor at a higher temperature. In our tests, annealing FBGs at 1000 °C for a few hundred hours reduced the wavelength drift at 900 °C to ~0.5 pm/h (0.03 °C/h), which, however, is still too high for accurate long-term temperature measurements. The same trend was reported in the literature when pre-annealing FBGs at 1100 °C and 1200 °C helped reduce wavelength drift at a lower temperature. The slow blue drift (0.1–0.5 pm/h rate) is more difficult to eliminate because of its elusive nature. However, it is probably possible to correct for the slow blue drift numerically as this phenomenon seems to be more predictable compared with the red drift. We would also like to emphasize that the Bragg wavelength drift at high temperatures should not affect FBG-based measurements performed on short time scales.
